# Comparison of Percutaneous Coronary Intervention Outcomes Among Patients With Obstructive Sleep Apnea, Chronic Obstructive Pulmonary Disease Overlap, and Pickwickian Syndrome (Obesity Hypoventilation Syndrome)

**DOI:** 10.7759/cureus.24816

**Published:** 2022-05-07

**Authors:** Rupak Desai, Sonali Sachdeva, Akhil Jain, Bisharah Rizvi, Hee Kong Fong, Jilmil Raina, Vikram Itare, Thomas Alukal, Anubhav Jain, Ankita Aggarwal, Gautam Kumar, Rajesh Sachdeva

**Affiliations:** 1 Division of Cardiology, Atlanta Veterans Affairs (VA) Medical Center, Decatur, USA; 2 Cardiovascular Section, Boston University School of Medicine, Boston, USA; 3 Internal Medicine, Mercy Catholic Medical Center, Darby, USA; 4 Internal Medicine, Saint Agnes Medical Center, Fresno, USA; 5 Cardiovascular Medicine, University of California Davis Medical Center, Sacramento, USA; 6 Medicine, Brookdale University Hospital and Medical Center, Brooklyn, USA; 7 Medicine, BronxCare Health System, Bronx, USA; 8 Medicine, Virginia Commonwealth University (VCU) Health Community Memorial Hospital, South Hill, USA; 9 Cardiology, Ascension Genesys Hospital, Grand Blanc, Michigan, USA; 10 Cardiology, Ascension Providence Hospital, Michigan, USA; 11 Division of Cardiology, Emory University School of Medicine/Atlanta Veterans Affairs (VA) Medical Center, Decatur, USA

**Keywords:** outcomes, chronic obstructive pulmonary disease overlap, obstructive sleep apnea, pickwickian syndrome, percutaneous coronary intervention

## Abstract

Background

Obstructive sleep apnea (OSA) is often present in coronary artery disease patients and confers a high risk of complications following percutaneous coronary interventions (PCI). The impact of two commonly associated comorbid conditions, chronic obstructive pulmonary disease (COPD) and obesity hypoventilation syndrome (OHS, Pickwickian syndrome) in OSA patients undergoing PCI has never been studied.

Methods

The National Inpatient Sample (NIS; 2007-2014) was queried using the International Classification of Diseases, Clinical Modification 9 (ICD-9-CM) codes to compare baseline characteristics, comorbidities, and outcomes in adults undergoing PCI with OSA, COPD-overlap syndrome, and OSA+OHS.

Results

Of a total of 4,792,177 PCI-related inpatient encounters, OSA, OSA-COPD overlap syndrome, and OSA+OHS were found to be present in 153,706 (median age 62 years, 79.4% male), 65135 (median age 65 years, 66.0% male), and 2291 (median age 63 years, 58.2% males) patients, respectively. The OHS+OSA cohort, when compared to the COPD-OSA and OSA cohorts, was found to have the worst outcomes in terms of all-cause mortality (2.8% vs. 1.5% vs. 1.1%), hospital stay (median 6 vs. 3 vs. 2 days), hospital charges ($147, 209 vs. $101,416 vs. $87,983). Complications, including cardiogenic shock (7.3% vs. 3.4% vs. 2.6%), post-procedural myocardial infarction (11.2% vs. 7.1% vs. 6.0%), iatrogenic cardiac complications (6.1% vs. 3.5% vs. 3.7%), respiratory failure, acute kidney injury, infections, and pulmonary embolism, were also significantly higher in patients with OHS+OSA. Adjusted multivariable analysis revealed equivalent results with OHS+OSA having worse outcomes than OSA-COPD and OSA.

Conclusion

Concomitant OHS and COPD were linked to worse clinical outcomes in patients with OSA undergoing PCI. Future prospective studies are warranted to fully understand related pathophysiology, evaluate and validate long-term outcomes, and formulate effective preventive and management strategies.

## Introduction

Obstructive sleep apnea (OSA) can worsen coronary atherosclerosis and is a predictor of symptomatic coronary artery disease (CAD) and subsequent adverse outcomes after percutaneous coronary interventions (PCI) [1-2]. OSA, at times, is accompanied by chronic obstructive pulmonary disease (COPD) and/or obesity hypoventilation syndrome (OHS). OSA and COPD coexist in an entity known as OSA-COPD overlap syndrome in the absence of any known confounders, other than smoking.

The prevalence of OHS (Pickwickian syndrome) in OSA patients ranges from 4$ to 50%, obesity being a confounding factor [3]. Both disorders individually are known to alter oxygenation, predisposing to hypercapnia. Hence, it could be summarized and hypothesized in this national population-based analysis that the effect of OHS and COPD on OSA would lead to a further deterioration of clinical outcomes in CAD patients undergoing PCI.

## Materials and methods

Data source and study population

The National Inpatient Sample (NIS) database (2007-2014) provides hospital administrative data under the Healthcare Research and Quality Healthcare Cost and Utilization Project (HCUP) [4]. Using the International Classification of Diseases, Clinical Modification 9 (ICD-9-CM) codes, this database was sought to identify baseline characteristics, in-hospital outcomes in patients with OSA or OSA-COPD overlap syndrome, and OSA-OHS undergoing PCI.

Outcomes

We studied and compared the effect of OSA, OSA-COPD overlap syndrome, and OHA-OSA on PCI hospitalizations. Reported outcomes include all-cause in-hospital mortality, complications like cardiogenic shock, postoperative myocardial infarction (MI), pericardial complications, iatrogenic cardiovascular complications (cardiac arrest/insufficiency or cardiorespiratory failure during and after or resulting from a procedure), kidney failure requiring dialysis, postoperative respiratory failure, and pulmonary embolism, a requirement of mechanical ventilation, length of in-hospital stay, hospital charges incurred, and disposition of the patient (routine, transfer to short-term hospital, transfer to other facilities, and home health care).

Statistical analysis

The Statistical Package for the Social Sciences (SPSS) version 24 (IBM Corp, Armonk, NY) was employed, and appropriate weighting and clustering techniques were applied as per the recommendations by HCUP to account for the complex survey design of the NIS database. The χ2 test was used for categorical variables. Normally distributed data were expressed as mean±SD and compared using independent t-tests. We performed multivariable analyses to evaluate the odds of post-PCI complications adjusting for demographics, hospital-level characteristics, and all baseline comorbidities. A p-value of <0.05 was considered statistically significant.

## Results

A total of 4,792,177 hospitalizations were identified for PCI having concomitant OSA (n=153,706, median age 62 years), OSA-COPD overlap syndrome (n=65,135, median age 63 years), or OSA-OHS (n=2,291, median age 65 years) between 2007 and 2014 (Table 1). Among all PCI-related admissions, OSA, OSA-COPD overlap, and OSA in combination with OSA were seen with a prevalence of 3.21%, 1.36%, and 0.05%, respectively.

**Table 1:** Baseline Characteristics and Outcomes of Hospitalizations for Percutaneous Coronary Intervention in Obstructive Sleep Apnea vs. OSA-COPD Overlap Syndrome vs. Obesity Hypoventilation Syndrome (n= 219,645) P-value < 0.05 indicates statistical significance. HMO=Health Maintenance Organization, SNF=Skilled Nursing Facility, ICF=Intermediate Care Facility, OSA=Obstructive Sleep Apnea, COPD=Chronic Obstructive Pulmonary Disease, OHS=Obesity Hypoventilation Syndrome, MI=Myocardial Infarction, PCI=Percutaneous Coronary Intervention, CABG=Coronary Artery Bypass Graft

Variable	Obstructive Sleep Apnea (n=153,706)		OSA-COPD Overlap Syndrome (n=65,135)			Obesity Hypoventilation Syndrome (n=804)			P
Age (years) at admission									<0.001
Mean age ± SD	62 (54-70)		65 (57-72)			63 (56-72)			
18-44	8,733	(5.7%)	2,060	(3.2%)	19		(2.4%)		
45-64	80,844	(52.6%)	30,354	(46.6%)	403		(50.1%)		
≥65	64,129	(41.7%)	32,721	(50.2%)	382		(47.5%)		
Sex									<0.001
Male	121,969	(79.4%)	42,982	(66.0%)	417		(51.8%)		
Female	31,722	(20.6%)	22,153	(34.0%)	387		(48.2%)		
Race									<0.001
White	107,697	(82.4%)	47,058	(83.0%)	605		(81.9%)		
African American	10,976	(8.4%)	5,329	(9.4%)	65		(8.8%)		
Hispanic	6,619	(5.1%)	2,349	(4.1%)	35		(4.7%)		
Asian or Pacific Islander	1,351	(1.0%)	370	(0.7%)	<11				
Native American	711	(0.5%)	307	(0.5%)	15		(2.0%)		
Other	3,298	(2.5%)	1,252	(2.2%)	15		(2.0%)		
Primary Expected Payer									<0.001
Medicare	72,184	(47.0%)	40,117	(61.7%)	485		(60.4%)		
Medicaid	7,661	(5.0%)	5,121	(7.9%)	65		(8.1%)		
Private, Including HMO	62,588	(40.8%)	15,782	(24.3%)	189		(23.5%)		
Self-Pay	5,594	(3.6%)	2,230	(3.4%)	25		(3.1%)		
No charge	463	(0.3%)	138	(0.2%)	<11				
Others	4,935	(3.2%)	1,628	(2.5%)	35		(4.3%)		
Non-Elective Admission	120,983	(78.9%)	53,061	(81.7%)	670		(83.4%)		<0.001
Bed Size of Hospital									
	12,887	(8.4%)	4,939	(7.6%)	76		(9.5%)		
	34,267	(22.4%)	15,409	(23.8%)	194		(24.1%)		
	105,532	(69.1%)	44,363	(68.6%)	534		(66.4%)		
Location/Teaching Status of Hospital									<0.001
Rural	8,171	(5.4%)	4,024	(6.2%)	63		(7.8%)		
Urban - Non-Teaching	53,144	(34.8%)	24,023	(37.1%)	312		(38.8%)		
Urban - Teaching	91,371	(59.8%)	36,663	(56.7%)	430		(53.4%)		
Region of Hospital									
Northeast	25,080	(16.3%)	9,499	(14.6%)	134		(16.7%)		
Midwest	48,682	(31.7%)	21,337	(32.8%)	158		(19.7%)		
South	56,664	(36.9%)	25,724	(39.5%)	332		(41.3%)		
West	23,280	(15.1%)	8,576	(13.2%)	179		(22.3%)		
Comorbidities									
Alcohol Abuse	2,661	(1.7%)	1,619	(2.5%)	15		(1.9%)		<0.001
Deficiency Anemias	17,529	(11.4%)	10,590	(16.3%)	109		(13.6%)		
Rheumatoid Arthritis/Collagen Vascular Diseases	3,184	(2.1%)	1,821	(2.8%)	<11				
Congestive Heart Failure	2,098	(1.4%)	1,832	(2.8%)	85		(10.5%)		
Coagulopathy	4,669	(3.0%)	2,544	(3.9%)	65		(8.1%)		
Depression	17,036	(11.1%)	9,333	(14.3%)	76		(9.5%)		<0.001
Diabetes, Uncomplicated	65,280	(42.5%)	30,263	(46.5%)	335		(41.7%)		<0.001
Diabetes With Chronic Complications	14,495	(9.4%)	7,422	(11.4%)	154		(19.1%)		<0.001
Drug Abuse	1,860	(1.2%)	1,114	(1.7%)	25		(3.1%)		<0.001
Hypertension	123,321	(80.2%)	51,931	(79.7%)	577		(71.7%)		<0.001
Hypothyroidism	16,630	(10.8%)	8,586	(13.2%)	75		(9.3%)		<0.001
Liver Disease	2,280	(1.5%)	1,182	(1.8%)	<11				<0.001
Fluid and Electrolyte Disorders	19,152	(12.5%)	11,636	(17.9%)	359		(44.6%)		<0.001
Peripheral Vascular Disorders	15,709	(10.2%)	10,958	(16.8%)	89		(11.1%)		<0.001
Pulmonary Circulation Disorders	603	(0.4%)	569	(0.9%)	25		(3.1%)		<0.001
Renal Failure	27,329	(17.8%)	14,840	(22.8%)	239		(29.7%)		<0.001
Smoking	53,288	(34.7%)	32,884	(50.5%)	148		(18.4%)		<0.001
Dyslipidemia	119,794	(77.9%)	46,936	(72.1%)	462		(57.4%)		<0.001
Prior MI/PCI/CABG	54,911	(35.7%)	24,711	(37.9%)	203		(25.3%)		
Valvular heart disease	495	(0.3%)	464	(0.7%)	15		(1.8%)		<0.001
Outcomes									
All-Cause In-Hospital Mortality	1,656	(1.1%)	987	(1.5%)	63		(7.9%)		<0.001
Disposition of Patient									<0.001
Routine	1,144	(0.7%)	444	(0.7%)	20		(2.5%)		
Transfer to Short-Term Hospital	5,899	(3.8%)	5,000	(7.7%)	159		(19.8%)		
Other Transfers (SNF, ICF, Another Facility)	8,655	(5.6%)	6,990	(10.7%)	135		(16.8%)		
Home Health Care	437	(0.3%)	240	(0.4%)	<11				
Length of Stay (Days) Mean ±SD	2 (1-4)		3 (2-6)			6 (3-13)			<0.001
Total Hospital Charges (Mean)	$59,499 (42,274- 87,983)		$67,325 (46,134- 101,416)			$113,845 (68,103-180,829)			<0.001

The study population majorly consisted of middle-old age (>45 years: ~95%), white (~80%), male (>60%) patients who were Medicare enrollees (>50% and above), admitted for non-elective reasons (~80%). Comorbidities like diabetes (47.7%) and its related complications (22.4%), congestive heart failure (6.0%), hypothyroidism (15.0%), liver disease (2.8%), pulmonary circulatory disorders (2.8%), and renal failure (35.1%) were most prevalent in the OSA-OHS group (p<0.001).

Concurrent OHS and OSA were associated with the worst outcomes, followed by OSA-COPD overlap syndrome and OSA alone (Table 2). All-cause inpatient mortality (2.8% vs 1.5% vs 1.1%), cardiogenic shock (7.3% vs 3.4% vs 2.6%), postoperative myocardial infarction (11.2% vs 7.1% vs 6.0%), iatrogenic cardiac complications (6.1% vs 3.5% vs 3.7%), hospital stay (median 6 vs 3 vs 2 days), and total incurred charges ($147,209 vs $101,416 vs $87,983) were higher in the OSA-OHS group, compared to the other two. Additionally, the OSA-OHS cohort showed higher rates of respiratory failure and mechanical ventilation, acute kidney injury, infections, pulmonary embolism, and lower rates of routine disposition after PCI than the OSA-COPD and OSA cohorts (all p-values 0.001). Adjusted multivariable analysis revealed equivalent results (Table 3) with OHS+OSA having worse outcomes than OSA-COPD, and OSA being referent.

**Table 2:** Outcomes of Percutaneous Coronary Intervention in OSA vs. OSA-COPD Overlap vs. OHS P-value < 0.05 indicates statistical significance. HMO=Health Maintenance Organization, SNF=Skilled Nursing Facility, ICF=Intermediate Care Facility, OSA=Obstructive Sleep Apnea, COPD=Chronic Obstructive Pulmonary Disease, OHS=Obesity Hypoventilation Syndrome

Outcomes	No OSA or OSA+COPD or OSA+OHS (N= 4,571,045)	OSA (N=153,706)	OSA+COPD Overlap Syndrome (N=65,135)	OSA+OHS (N=2,291)	P
All-cause In-hospital mortality	1.9%	1.1%	1.5%	2.8%	<0.001
Complications					
Cardiogenic shock	3.4%	2.6%	3.4%	7.3%	<0.001
Postoperative myocardial infarction	5.9%	6.0%	7.1%	11.2%	<0.001
Iatrogenic cardiac complications including cardiac arrest	3.8%	3.7%	3.5%	6.1%	<0.001
Pericardial complications	0.6%	0.5%	0.7%	0.7%	<0.001
Postoperative respiratory failure	0.6%	0.7%	1.3%	2.8%	<0.001
Postoperative pulmonary embolism	0.2%	0.3%	0.3%	0.4%	<0.001
Perioperative stroke	0.7%	0.6%	0.6%	0.6%	<0.001
Postoperative infection	1.1%	0.9%	1.7%	3.5%	<0.001
Acute kidney injury requiring dialysis	0.3%	0.5%	0.6%	1.9%	<0.001
Mechanical circulatory support	3.7%	3.0%	3.6%	6.5%	<0.001
Disposition of Patient					<0.001
Routine	87.0%	88.4%	79.0%	56.5%	
Transfer to a short-term hospital	0.9%	0.7%	0.7%	1.1%	
Other Transfers (SNF, ICF, others)	4.5%	3.8%	7.7%	20.9%	
Home Health Care	5.3%	5.6%	10.7%	18.6%	
Length of stay (days); median (IQR)	2 (1-4)	2 (1-4)	3 (2-6)	6 (4-10)	<0.001
Hospital charges (USD); median (IQR)	56,027 (39,103-83,689)	59,499 (42,274-87,983)	67,325 (46,134-101,416)	98,083 (65,733-147,209)	<0.001

**Table 3:** Multivariable Odds of Complications in Patients Undergoing PCI With OSA-COPD Overlap and OSA+OHS as Compared to OSA Alone: Adjusted OR (95% CI:LL-UL) P-value < 0.05 indicates statistical significance. OR=Adjusted Odds Ratio, CI=Confidence Interval, LL=Lower Limit, UL=Upper Limit, OSA=Obstructive Sleep Apnea, COPD=Chronic Obstructive Pulmonary Disease, OHS=Obesity Hypoventilation Syndrome

	OSA	OSA+COPD Overlap	P	
All-cause in-hospital mortality	Referent	1.21 (1.11-1.32)	<0.001	
Cardiogenic shock	Referent	1.25 (1.18-1.33)	<0.001	
Postoperative myocardial infarction	Referent	1.06 (1.02-1.11)	0.005	
Iatrogenic cardiac complications	Referent	1.02 (0.97-1.08)	0.413	
Postoperative respiratory failure	Referent	1.77 (1.59-1.96)	<0.001	
Postoperative infection	Referent	1.51 (1.37-1.65)	<0.001	
Acute kidney injury requiring dialysis	Referent	1.34 (1.29-1.39)	<0.001	
Mechanical circulatory support	Referent	1.18 (1.11-1.25)	<0.001	

## Discussion

To the best of our knowledge, this is the first study reporting short-term PCI outcomes in patients suffering from OSA alone and combined with disorders like COPD and Pickwickian syndrome. Our study considered the 2007-2014 inpatient sample and revealed that OSA combined with OHS and COPD predicted significantly worse post-PCI outcomes compared to OSA alone. Adverse outcomes incorporated inpatient mortality (2.8% vs. 1.5% vs. 1.1%), prolonged hospital stays (median 6 vs. 3 vs. 2 days), hospital charges ($147,209 vs. $101,416 vs $87,983), frequent cardiovascular complications like cardiogenic shock (7.3% vs. 3.4% vs. 2.6%), postoperative MI (11.2% vs 7.1% vs 6.0%), post-procedural respiratory failure (2.8% vs. 1.3% vs. 0.7%), acute kidney injury (1.9% vs. 0.6% vs 0.5%), and infection (3.5% vs. 1.7% vs. 0.9%). On adjusted multivariable analysis, the OSA+OHS cohort was more likely to suffer from post-procedural complications or death due to any cause than the OSA-COPD or OSA cohorts.

The presence of OSA is found to be associated with a risk of recurrent major adverse cardiac events (MACE), all-cause and cardiac deaths, and repeat revascularization following PCI [5]. It is commonly seen in older adults and more so in patients who have CAD with a prevalence of 38%-65% [6].

OHS is an amalgamation of obesity, daytime hypercapnia (PaCO2 >45 mm hg), and sleep-disordered breathing in the absence of other causes of hypoventilation. Although OSA and OHS are similar in their clinical presentation (snoring, breathing sleep disorder, and daytime sleepiness), OSA does not commonly result in hypoventilation and hypercapnia, unlike OHS. The latter is a clinically more severe entity, resulting in a higher daytime PaCO2, chronic respiratory failure, pulmonary hypertension, enlarged right ventricle, systemic hypertension, and an overall complicated disease course [7-9]. Data suggest that 90% of OHS patients also suffer from co-existing OSA [10]. This coexistence can potentially worsen things, as revealed by a study that reported higher PaC02 levels and unfavorable respiratory indices compared to OSA patients [11].

The prevalence of COPD in OSA and vice versa is sometimes seen to be unexpectedly high, with the strongest data reported by the Sleep Heart Health Study [12]. Compared to patients with OSA alone, those suffering from the overlap syndrome tend to be older, have higher mean pulmonary arterial pressure, and are often hypercapnic and hypoxemia [13]. As seen in both OHS and OSA, sleep-disordered breathing could affect outcomes after PCI by causing a larger infarct area and impaired healing, as reported by Buchner and colleagues [14]. Adding to the postoperative risk is the concomitant presence of metabolic and cardiovascular co-morbidities in OHS patients, frequently diagnosed before the diagnosis of OHS. According to a study comparing OHS and OSA, OHS patients had a higher mortality rate, and the majority of deaths were attributable to fatal cardiovascular events [15]. The major contributing factor to adverse cardiovascular outcomes is the intermittent hypoxia triggered by repetitive episodes of apnea and hypopnea seen in OSA patients [16].

The combined presence of COPD and severe OSA defined by the Apnea-Hypopnea index (AHI) or degree of nocturnal hypoxemia is associated with adverse cardiovascular events and mortality [17]. The presence of obesity and airway obstruction in OHS and COPD respectively compound the limitation of inter-apneic ventilation in OSA, possibly exacerbating hypercapnia and worsening post-procedural respiratory failure. 

OHS patients are at an increased risk of developing heart failure, ventricular dysfunction, cor pulmonale, pulmonary hypertension, and angina pectoris than obese eucapnic individuals [18]. A study found that the prevalence of left ventricular dysfunction was high in OHS patients even with mild to moderate OSA [19]. Prior studies reveal that OHS patients have a high prevalence of right ventricular dysfunction, left ventricular hypertrophy, arrhythmias, and pulmonary hypertension due to a reflex elevation of arterial blood pressure and increased heart workload following chronic hypoxia [20]. These changes in cardiac function could contribute to significantly higher odds of cardiovascular complications and the requirement of hemodynamic support post-PCI in patients with OHS and COPD overlap as compared to OSA alone.

The limitations of this analysis should be considered before drawing any firm conclusion. First, this retrospective study based on administrative ICD-9 CM coding runs the risk of under as well as overreporting outcomes and limits any causal association. Secondly, this analysis only reports in-patient outcomes as we did not have outpatient data, long-term follow-up data, or functional outcomes. Further, the results of arterial blood gas analysis, laboratory investigations, medication history, and echocardiographic or electrophysiological findings were not available. Moreover, the severity of OSA, OSA-COPD overlap, and OHS was not known before PCI. Although multivariable regression analysis was controlled for all potential confounding factors, residual confounding effects cannot be overlooked due to the retrospective nature of the study and variation in access, preference, and level of care provided among different hospitals.

## Conclusions

Although OHS remains underdiagnosed globally, it can predict adverse in-hospital outcomes when presented with OSA as shown in our study. With the aggravation of OHS by conditions like COPD and OSA, complications tend to merge, resulting in worse outcomes after cardiac interventions. Such high-risk patients may require cardiorespiratory monitoring, leading to a prolonged hospital stay and higher healthcare costs. Future prospective studies are warranted to fully understand related pathophysiology, evaluate and validate long-term outcomes, and formulate effective preventive and management strategies.
